# Corticotrophin-releasing hormone binding protein in the basolateral amygdala: divergent roles in cocaine and opioid addiction like behaviors

**DOI:** 10.1016/j.addicn.2025.100221

**Published:** 2025-08-05

**Authors:** Courtney P. Wood, Arnav Gurha, Caitlin Crook, Angelica Martinez, Selen Dirik, Cloe Moreno, Anirudh Vaiyapuri, Avraham Libster, Francesca Telese, Giordano de Guglielmo

**Affiliations:** a Psychiatry Department, School of Medicine, University of California, San Diego, San Diego, CA 92093, USA; b School of Pharmacy, Pharmacology Unit, University of Camerino, Camerino (MC), 62032, Italy; c Department of Neurobiology and Behavior, University of California, Irvine, Irvine, CA 92697, USA

**Keywords:** Amygdala, Addiction, Cocaine, Oxycodone, Corticotrophin-releasing binding protein

## Abstract

Cocaine and oxycodone use disorders represent urgent public health burdens, with limited treatment options for opioid dependence and no approved pharmacotherapies for cocaine-related disorders, highlighting the critical need to uncover novel molecular targets in the addiction neurocircuitry. The basolateral amygdala (BLA) modulates withdrawal and drug-seeking behaviors in substance use disorders (SUDs), but the molecular drivers of these effects are poorly understood. Here, we investigated the role of corticotrophin-releasing hormone binding protein (CRHBP) in the BLA using viral-mediated knockdown, single-nucleus RNA sequencing (snRNA-seq), and operant self-administration in rats. snRNA-seq revealed *Crhbp* expression in BLA somatostatin-positive interneurons, with minimal presence in the central amygdala. *Crhbp* knockdown in the BLA decreased cocaine self-administration and cue-induced reinstatement, suggesting a role in sustaining stimulant addiction. Conversely, it increased oxycodone intake and progressive ratio breakpoints, indicating heightened opioid reinforcement, while also elevating cue-induced reinstatement after 24 h of abstinence, reflecting enhanced opioid-seeking. These opposing effects highlight *Crhbp’s* substance-specific influence on addiction-related behaviors in the BLA. Our findings position *Crhbp* as a potential therapeutic target gene for tailored interventions in cocaine and opioid SUDs.

## Introduction

Substance use disorders (SUDs) involving cocaine and oxycodone constitute a pressing global health crisis, affecting at least 15 million people worldwide [1]. In the United States, cocaine remains a leading illicit drug of abuse [2], with overdose deaths surging sevenfold over the past two decades [3]. Concurrently, oxycodone, a widely misused prescription opioid, contributes significantly to overdose mortality, surpassing heroin-related deaths since 2000 [4,5]. Despite FDA-approved treatments like methadone and buprenorphine for opioid use disorder (OUD), their efficacy and accessibility remain limited, while no pharmacotherapies exist for cocaine use disorder [6,7]. This therapeutic gap, compounded by stigma and low treatment uptake [8], underscores the urgent need to identify novel molecular targets that address the negative reinforcement driving drug-seeking across SUDs, rather than relying solely on abstinence as a treatment endpoint.

Cocaine and oxycodone exert their reinforcing effects through distinct yet overlapping neurobiological mechanisms within the brain’s reward system. Cocaine blocks the dopamine transporter (DAT), amplifying dopamine signaling in the nucleus accumbens (NAc) to produce euphoria and heightened arousal [9,10]. Oxycodone, by contrast, activates μ-opioid receptors (MORs) in regions like the ventral tegmental area (VTA), disinhibiting dopaminergic projections to the NAc and eliciting analgesia alongside reward [11,12]. Despite these differences, both drugs engage overlapping circuits, including the amygdala, which regulates negative emotional states, such as anxiety, irritability, and dysphoria, during withdrawal [13]. These states perpetuate drug-seeking and reinstatement in preclinical models of cocaine and oxycodone use [14,15], suggesting a shared role for amygdalar circuits in sustaining SUDs across drug classes.

Within the amygdala, the basolateral amygdala (BLA) is a pivotal hub for processing reward-related information and forming drug-associated memories, driving cue-induced drug-seeking behaviors critical to addiction [16]. Lesions or inactivation of the BLA disrupt these cue-reward associations, reducing relapse-like behaviors in preclinical models [17–19]. Complementing this, the corticotropin-releasing factor (CRF) system, a key regulator of stress responses, mediates the negative affective states during withdrawal that precipitate relapse, particularly through its actions in the extended amygdala, including the BLA [20]. Notably, the CRH binding protein (CRHBP, encoded by the *Crhbp* gene), which modulates CRF signaling [21], has emerged as a potential modulator of addiction-related behaviors [22,23].

Recent advances in single-nucleus RNA sequencing (snRNA-seq) have illuminated the cellular and molecular heterogeneity of reward-related brain regions, including the amygdala [24]. Our prior work applied snRNA-seq and snATAC-seq to amygdala tissue from cocaine-dependent outbred rats after prolonged intravenous self-administration, revealing transcriptional signatures of escalation, motivation, and compulsive drug-seeking [25]. Differential expression analysis revealed that *Crhbp* was downregulated in rats with high versus low addiction-like behaviors, specifically in somatostatin-positive (Sst+) interneurons (as shown in Supplementary Table S7 from [25]). This finding aligns with reports of CRHBP’s role in modulating alcohol-related behaviors, where its knockdown in the central amygdala (CeA) reduced ethanol intake in dependent rats [22,23]. In humans, a *CRHBP* variant is associated with cocaine cessation success during OUD treatment [26], though its link to opioid dependence remains inconsistent [27]. These mixed findings suggest *Crhbp*’s involvement in SUDs may be region- and substance-specific.

To better understand its potential as a pivot point in substance-specific neurocircuitry, we sought to investigate the role of *Crhbp* in both cocaine and oxycodone self-administration and reinstatement. We leveraged snRNA-seq from the CeA and BLA of naïve rats to investigate its tissue- and cell type-specific expression. Then, we knocked down its expression specifically in the BLA of rats with an AAV/DJ8 virus carrying a shRNA against *Crhbp*, followed by intravenous self-administration of cocaine or oxycodone and cue-induced reinstatement assays. Our results reveal opposing effects, attenuated stimulant-related behaviors versus enhanced opioid reinforcement and seeking, offering new insights into BLA-mediated mechanisms of SUDs and positioning *Crhbp* as a candidate gene for targeted therapeutic development.

## Materials & methods

### Animals

Males and Females Wistar rats (*n* = 36, 18 males and 18 females, Charles River Laboratories, Wilmington, MA, USA) were shipped at 3–4 weeks of age, kept in quarantine for 1 week, and then housed two per cage on a 12 hr/12 hr reversed light/dark cycle (lights off at 8 am, for cocaine studies) and a 12 hr/12 hr standard light/dark cycle (lights off at 5 pm, for oxycodone studies) in a temperature (20–22 °C) and humidity (45–55 %) controlled vivarium with ad libitum access to tap water and standard rodent food pellets. All procedures were conducted in strict adherence to the National Institutes of Health Guide for the Care and Use of Laboratory Animals and were approved by the Institutional Animal Care and Use Committee of UC San Diego (S21145).

### Single nuclei RNA-seq analysis

The count matrix files for the rat CeA and BLA snRNA-seq datasets were downloaded from [25], in which for each tissue, brain punches from 3 rats were pooled to generate the single-nucleus libraries (snRNA-seq and snATAC-seq). In the present study, only the snRNA-seq data were analyzed. The processing and data analysis of these snRNA-seq data was performed in python (v3.10.16) using Scanpy (v1.10.4) [28]. Nuclei with high ambient RNA expression were removed using CellBender (v0.3.0) [29] with a cell probability threshold of 0.5. This led to a reduction in number of nuclei from 4792 to 2993 nuclei for the BLA dataset and a decrease from 18,508 to 12,084 nuclei in the CeA dataset. Additionally, the percentage of mitochondrial transcripts were examined and nuclei expressing >20 % were filtered out. This led to a further reduction from 2993 nuclei to 2990 nuclei for the BLA dataset and a decrease from 12,084 nuclei to 11,796 in the CeA dataset. Further quality control was applied by filtering low-quality cells expressing <100 genes and genes detected in fewer than 3 cells. For the BLA dataset, the number of nuclei remained unchanged after this step, however, the number of genes reduced from 25,399 to 16,901. In the CeA dataset, this step reduced the number of nuclei from 11,796 to 11, 270 nuclei and reduced the number of genes from 25,399 to 18,367 genes. Doublet detection was then performed using the scrublet [30] algorithm with parameters sim doublet_ratio=2.0 and expected_doublet_ratio=0.05; no doublets were detected.

Next, the data was normalized using the *normalize total* function (target_sum=1e6 per cell) followed by log transformation using log1p. The top 2000 most variable genes were identified using the *highly_variable_genes* function to capture the genes driving cell-to-cell heterogeneity. For dimensionality reduction, UMAP was applied, followed by the leiden clustering algorithm at 0.50 resolution, which yielded 9 clusters and 12 clusters for the BLA and CeA datasets, respectively. Each cluster was manually annotated using a set of well-established broad cell type marker genes. In the BLA dataset, clusters 0 and 1 were excluded due to ambiguous markers, and cluster 3 was excluded due to a lack of cell-type marker expression, reducing the number of nuclei to 1156. In the CeA dataset, clusters 0 and 1 were excluded from subsequent analysis due to ambiguous marker expression, reducing the number of nuclei to 8479. To achieve further granularity in the annotations, the top 20 most variable genes within each cluster were examined to identify specific marker genes. These refined labels and the processed data were used to generate the final visualization.

We also used snRNA-seq data from HS rats with high and low addiction index from [25] to generate a violin plot showing differential gene expression for *Chrbp* in amygdala cell types. Bootstrapping differential expression estimates. To generate bootstrap distributions of Crhbp differential expression effect sizes, we resampled nuclei with replacement 1000 times, separately for nuclei from high and low addiction index (AI) rats, thereby preserving the sample size of each group. For each bootstrap iteration, we performed negative binomial regression using Seurat’s FindMarkers() function and recorded the coefficients for the high and low AI conditions. These coefficients were then rescaled to log_2_ fold-change (log_2_FC) units. This approach produces slightly different estimates compared to Seurat’s reported average (avg)_log_2_FC, as the bootstrap method does not add a pseudocount and accounts for covariates. The resulting distribution of bootstrap log_2_FC estimates was visualized with violin plots overlaid with box-and-whisker plots.

### RNAscope and image acquisition

Rat brains were flash-frozen, and 20 μm coronal sections were obtained using a cryostat. Brain sections (bregma point −2.64 AP) containing the amygdala were selected for RNA-FISH. RNA-FISH was performed using the RNAscope Multiplex Fluorescent Reagent Kit v2 (ACD Bio, 323,100) following the user manual (ACD Bio, USM-323,100). We used the following probes for Rn-Crhbp (ACD Bio, 574,501), Rn-Sst (ACD Bio, 412,181-C4) and Rn-Slc32a1 (ACD Bio, 424,541-C2). We used fluorophores Opal 570, Opal 690, and Opal 520, included in the Opal 3-plex detection kit (Akoya Bioscience, NEL810001KT). All images were acquired using the Keyence BZ-X810 All-in-One wide-field fluorescence microscope. Z-stacks of the amygdala were obtained at 20x magnification using an air immersion objective lense (PlanFluor 20x, NA 0.45; Nikon). Fluorophores were selectively excited using ET-DAPI (CHROMA, 49,000), EGFP (CHROMA, 49,002), ET-CY3/R (CHROMA, 49,004), or ET-CY5.5 (CHROMA, 49,022) filter sets.

### Viral construct generation

AVV plasmid constructs were designed and cloned using Vector-Builder, and their maps and validated sequences can be retrieved using the following vector IDs: Scramble shRNA AAV vector ID is VB180117–1020znr, and shRNA-Crhbp AAV vector ID is VB900003–4285vht. Both vectors included an EGFP reporter gene. Vectors were packaged into viruses by the Gene Transfer, Targeting and Therapeutics Viral Vector Core at the Salk Institute for Biological Studies.

### Virus injection

Rats were deeply anesthetized with vaporized isoflurane (2–5 %) and placed into a stereotax. To reach the basolateral amygdala (BLA), the following coordinates were used with reference to bregma: anterior/posterior, −2.8 mm; medial/lateral, ±5.1 mm; and dorsal/ventral, −8.5 mm. AAV/DJ8-shCrhbp2 or AAV/DJ8-shControl stock viruses were diluted to a titer of 9.90 e^12^ gc/mL and infused at a rate of 100 nL per minute for 10 min, resulting in final infusion volume of 1 μL. The animals were allowed to recover for 2 weeks after surgery.

### Intravenous catheterization

Exactly two weeks after viral injection surgeries, rats were implanted with intravenous jugular catheters. Animals were anesthetized with vaporized isoflurane (2–5 %) and intravenous catheters were aseptically inserted into the right jugular vein using the procedure described previously [31]. Catheters consisted of Micro-Renathane tubing (18 cm, 0.023-inch inner diameter, 0.037-inch outer diameter; Braintree Scientific, Braintree, MA, USA) attached to a 90-degree angle bend guide cannula (Plastics One, Roanoke, VA, USA), embedded in dental acrylic, and anchored with mesh (1 mm thick, 2 cm diameter). Tubing was inserted into the vein following a needle puncture (22 G) and secured with a suture. The guide cannula was punctured through a small incision on the back. The outside part of the cannula was closed off with a plastic seal and metal cover cap, which allowed for sterility and protection of the catheter base. Flunixin (2.5 mg/kg, s.c.) was administered as analgesic, and Cefazolin (330 mg/kg, i.m.) as antibiotic. Rats were allowed one week for recovery prior to any self-administration. They were monitored and flushed daily with heparinized saline (10 U/ml of heparin sodium; American Pharmaceutical Partners, Schaumberg, IL, USA) in 0.9 % bacteriostatic sodium chloride (Hospira, Lake Forest, IL, USA) that contained 52.4 mg/0.2 ml of Cefazolin. At the conclusion of the study, catheters were tested for patency with an infusion of a methohexital/saline solution (5 mg/kg). Rapid loss of muscle tone was interpreted as an indication of patency. Any animal that failed to react to the infusion was excluded from the study.

### Drug preparation and administration

Cocaine hydrochloride (National Institute on Drug Abuse, Bethesda, MD, USA) was dissolved in 0.9 % sterile saline to yield a dose of 0.5 mg/kg/infusion. This dose was selected based on previous literature from our lab and others’ demonstrating that this dose is commonly used in rat self-administration studies and is effective in producing addiction-like behaviors [32–34]. This dose has been shown to maintain stable responding and induce escalation of intake, motivation, and compulsive-like responding in a significant proportion of animals [33].

Oxycodone (Sigma-Aldrich, St Louis, MO, USA) was dissolved in 0.9 % sterile saline to yield a dose of 0.15 mg/kg/infusion. This dose was selected based on previous literature from our lab and others’ demonstrating that this dose is commonly used in rat self-administration studies and is effective in producing addiction-like behaviors. This dose has been shown to maintain stable responding and induce escalation of intake, motivation, and compulsive-like responding in a significant proportion of animals [31,35,36]. To ensure consistent dosing for both cocaine and oxycodone, animals were weighed weekly to adjust the drug solution concentration, rounded to the nearest ten grams.

### Operant self-administration

Self-administration (SA) was performed in operant conditioning chambers (29 cm × 24 cm × 19.5 cm; Med Associates, St. Albans, VT, USA) that were enclosed in lit, sound-attenuating, ventilated environmental cubicles. The front door and back wall of the chambers were constructed of transparent plastic, and the other walls were opaque metal. Each chamber was equipped with two retractable levers that were located on the front panel. Each session was initiated by the extension of two retractable levers into the chamber. Cocaine hydrocholoride (0.5 mg/kg/infusion) or oxycodone (0.15 mg/kg/infusion) were delivered through plastic catheter tubing that was connected to an infusion pump. Drug availability was signaled by an intermittent tone (7 kHz, 70 dB), present throughout the session, which served as a discriminative stimulus. The infusion pump was activated by responses on the right (active) lever that were reinforced on a fixed ratio (FR) 1 schedule, with the delivery of 0.1 mL of the drug per lever press over 6 s for cocaine, 3.5 s for oxycodone followed by a 20 s timeout period that was signaled by the illumination of a cue light above the active lever, during which active lever presses did not result in additional infusions. The right lever was consistently designated as the active lever to maintain stable cue associations across training and cue-induced drug-seeking sessions, avoiding potential confounds from pseudorandom lever variation. Responses on the left inactive lever were recorded but had no scheduled consequences. Fluid delivery and behavioral data recording was controlled by a computer with the MED-PC IV software installed. Rats were trained to self-administer cocaine in 20 long access (LgA) sessions (6 hr/day, 5 days/week). Rats were trained to self-administer oxycodone in 12 overnight long access (LgA) sessions (12 hr/day, 5 days/week). Self-administration sessions took place during the dark phase of the light cycle. At the end of the LgA phase the oxycodone-dependent animals then underwent progressive ratio testing, followed by extinction and reinstatement testing.

### Progressive ratio testing

Rats were tested on a progressive ratio (PR) schedule of reinforcement in which the response requirements for receiving a single reinforcement increased according to the following 1, 2, 4, 6, 9, 12, 15, 20, 25, 32, 40, 50, 62, 77, 95, 118, 145, 178, …. The breakpoint was defined as the last ratio attained by the rat prior to a 60 min period during which a ratio was not completed, which ended the experiment.

### Cue-induced drug seeking

Cue-induced drug seeking behavior was assessed following abstinence periods of 24 h, 1 week, or 4 weeks after the final long-access (LgA) self-administration session for cocaine or oxycodone. Drug seeking sessions occurred at the same time of day as the preceding LgA sessions and were conducted in the same operant conditioning chambers. During these sessions, the intermittent tone (7 kHz, 70 dB) that served as a discriminative stimulus for drug availability during self-administration was presented throughout, and responses on the previously active (right) lever triggered the illumination of the cue light above the lever for 20 s, mimicking the timeout signal from training. However, no drug infusions were delivered. Active lever presses were recorded as a measure of drug-seeking behavior, while inactive lever responses were logged but had no programmed consequences. Each drug seeking session lasted 2 h.

### RT-PCR

In a separate cohort of animals (*n* = 16), the virus was administered as described in *Virus Injection* and allowed to express for 3 weeks. After 3 weeks, animals were euthanized by CO_2_ overdose and decapitation. Whole brain was collected and the BLA was isolated by microdissection and stored in RNA*later* stabilization solution (ThermoFisher Scientific, Carlsbad, CA, USA) at −20 °C until processing. Total RNA from BLA tissue was extracted using an RNeasy kit (Qiagen, Valencia, CA, USA) and first-strand cDNA was generated using M-MLV reverse transcriptase (Invitrogen, Carlsbad, CA, USA). Areas used for tissue collection and processing were sanitized with 70 % ethanol solution then treated with RNAse inhibitor (RNAse Out, G-Biosciences, St. Louis, MO, USA). Reverse transcription of total RNA was performed with random hexamers (Invitrogen, Carlsbad, CA, USA) for 50 min at 37 °C. qRT-PCR was carried out using PrimePCR Assays (Biorad, Irvine, CA, USA) with primers for corticotrophin releasing hormone binding protein (Crhbp) and Glyceraldehyde 3-phosphate dehydrogenase (Gapdh, housekeeping gene) using the preconfigured Sybr green assay (Biorad, Irvine, CA, USA). Reactions were run in triplicate values expressed as relative mRNA expression. Gapdh was selected as a housekeeping gene for experimental conditions; no changes in its expression were found across conditions included in our analysis.

### Experimental design & statistical analysis

Details regarding the experimental design of individual experiments are provided in the figure legends. Data were analyzed by GraphPad Prism version 9.5.0 (GraphPad Software, La Jolla, CA, USA) using unpaired Student’s *t*-tests (two-tailed), One sample *t*-tests, one-way ANOVA, or two-way ANOVA, with Bonferroni’s multiple comparisons *post-hoc* test when appropriate. Results are expressed as means ± *S*.E.M. and significance was determined at *p* < 0.05.

## Results

### Crhbp is expressed in Sst+ interneurons of the BLA

**Fig. 1.**
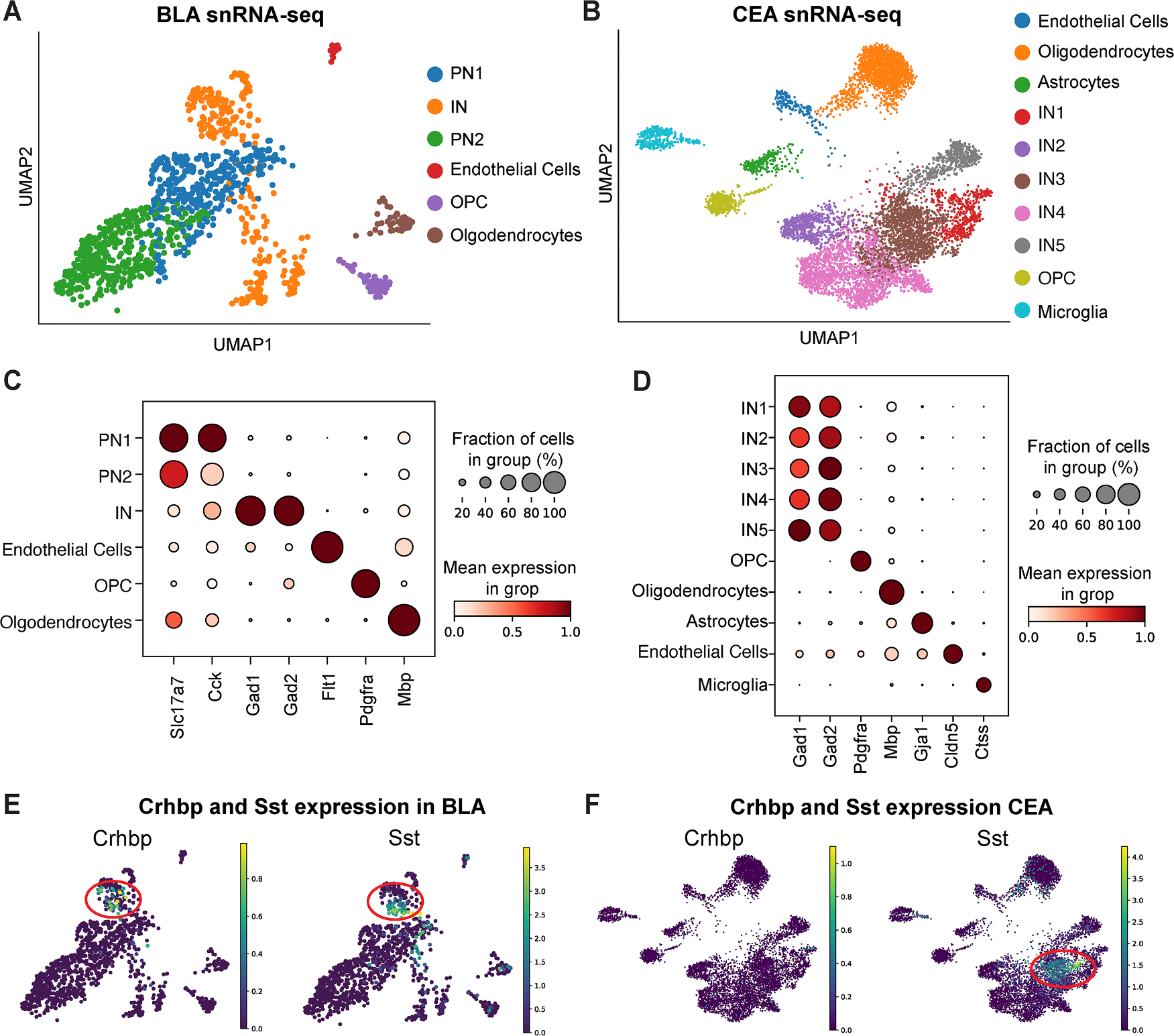
*Crhbp* expression in the BLA and CeA. (A) UMAP projection of the BLA dataset (*n* = 1156 nuclei). Each point represents a single nucleus, and clusters are color-coded by their annotated cell types. (B) UMAP projection of the CeA dataset (*n* = 8479 nuclei). Clusters are similarly color-coded by annotated cell types. (C) Dot plot visualization of select marker genes across annotated clusters of the BLA dataset. The size of the dot indicates the fraction of nuclei within that cluster expressing the gene, whereas the color intensity reflects the mean expression level (log1p-transformed). (D) Dot plot visualization of select marker genes across annotated clusters of the CeA dataset. The size of the dot indicates the fraction of nuclei within that cluster expressing the gene, whereas the color intensity reflects the mean expression level (log1p-transformed). (E) UMAP visualization of the BLA nuclei color-coded by the relative count normalized (log1p) expression of *Crhbp* (left) and *Sst* (right). Warmer colors indicate higher expression levels. (F) UMAP visualization of CeA nuclei color-coded by the relative count normalized (log1p) expression of *Crhbp* and *Sst*.

*Crhbp* was identified as differentially expressed gene in the Sst+ interneurons of the amygdala from rats with high cocaine addiction-like behavior compared to those with low addiction-like behavior ([25] Supplementary Fig. 1A). To further investigate the expression of *Crhbp* in distinct anatomical regions of the rat amygdala, including the BLA and CeA, we analyzed previously published snRNA-seq datasets from each brain region of 3 naïve rats [25]. After ambient RNA correction and filtering out low-quality nuclei, lowly expressed genes and doublets (Supplementary Fig. 1B–C), we identified 1156 high-quality nuclei in the BLA and 8479 in the CeA, which were used for subsequent clustering. Cell-type annotation was performed by comparing cluster-specific marker genes to known neuronal and non-neuronal markers (Supplementary Fig. 2A, B). In agreement with the known cell type composition of these brain regions, most nuclei (52 %) in the BLA were annotated as excitatory principal neurons (PN) expressing *Scl17a7* and *Cck*, while most nuclei (87 %) in the CeA were annotated as inhibitory neurons (IN) expressing *Gad1* and *Gad2* (Supplementary Fig. 2C, D). In the BLA, we identified two PN subtypes, one IN subtype, and glial cell types, including *Pdgfra*+ oligodendrocyte precursor (OPC) cells, *Mbp*+ oligodendrocytes, and *Ftl1*+ endothelial cells (Fig. 1A, C). In contrast, the neuronal populations in the CeA included five IN subtypes, along with *Gja1*+ astrocytes, *Pdgfra*+ OPC, *Mbp*+ oligodendrocytes, *Cldn5*+ endothelial cells, and *Ctss*+ microglia (Fig. 1B, D).

Further examination of gene expression patterns showed that *Crhbp* was expressed in the BLA in 45 of the 275 IN cells that co-expressed *Sst* (Fig. 1E, Supplementary Figure 2E). In contrast, CeA neurons did not exhibit significant *Crhbp* expression, while clearly expressing *Sst* in IN3 subtype (Fig. 1F). Taken together, these data indicate that *Crhbp* expression is restricted to a subset of *Sst*+ interneurons in the BLA, suggesting a unique role for *Crhbp* in modulating local inhibitory circuits within the BLA.

### Crhbp is downregulated in the BLA of rats following AAV-shRNA-mediated knockdown

**Fig. 2.**
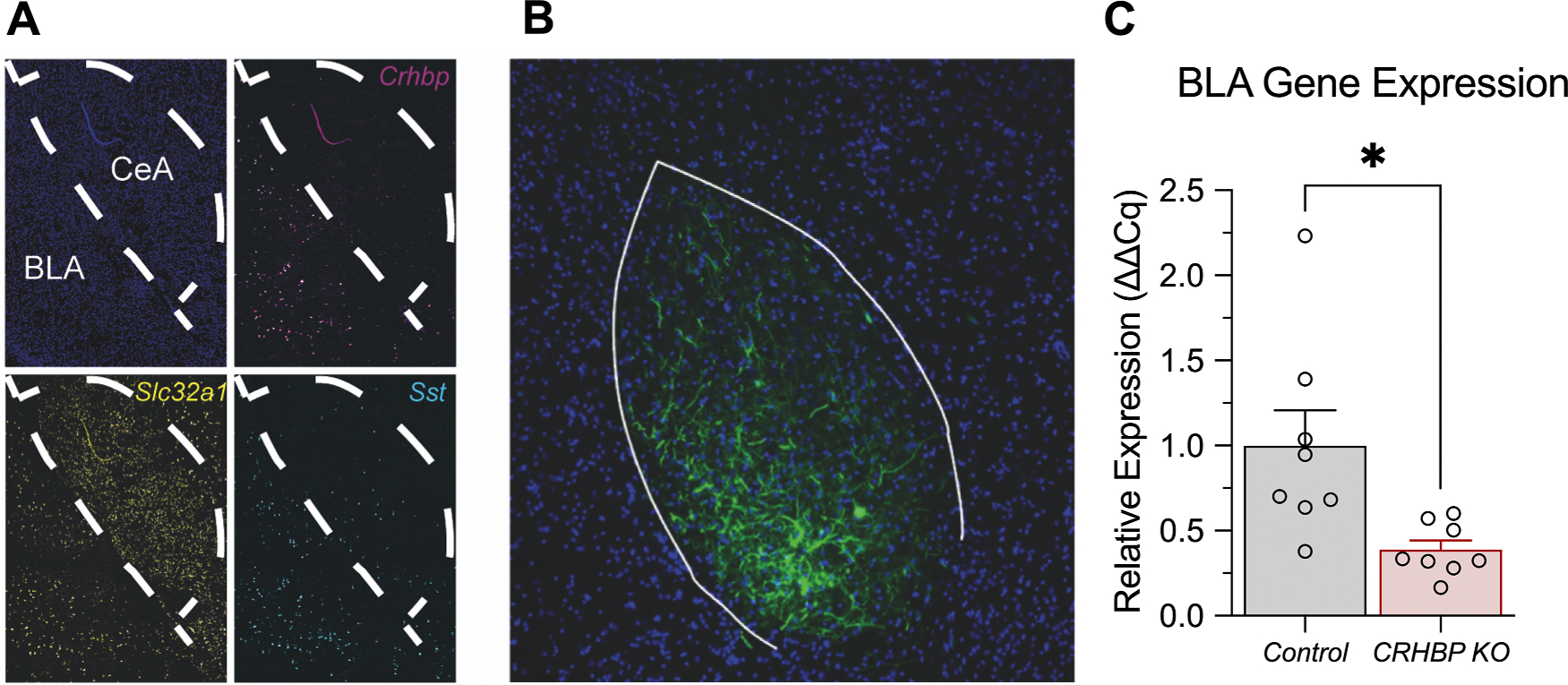
Validation of CRHBP Knockdown. (A) Representative images for the RNA fluorescent in situ hybridization (FISH) showing expression of *Crhbp, Slc32a1*, and *Sst* in the BLA and CeA. (B) Representative image showing GFP expression in the BLA after injection of AVV-shCrhbp by stereotaxic surgery. Bregma point −2.64 AP; scale bar 200 um. (C) RT-PCR showing *Crhbp* expression is significantly reduced in the BLA of *Crhbp* KD animals compared to controls. Data presented as mean ± SEM..*p < *0.05* vs. Control.

To validate our snRNA-seq findings, we conducted RNAscope in situ hybridization on coronal amygdala sections from rats (Fig. 2A). This confirmed *Crhbp* expression predominantly in the basolateral amygdala (BLA), where it co-localized with interneuron markers *Sst* (somatostatin) and *Slc32a1* (VGAT), consistent with the transcriptomic data. In contrast, minimal *Crhbp* signal was detected in the central amygdala (CeA), reinforcing its region-specific expression. To confirm *Crhbp* knockdown efficacy, we performed RT-qPCR on BLA tissue from a separate cohort of rats that received AAV/DJ8-shCrhbp (*n* = 8) or AAV/DJ8-shControl (*n* = 8) injections but did not undergo intravenous drug administration. Relative *Crhbp* mRNA expression in the BLA of shCrhbp-injected rats was significantly reduced compared to shControl-injected rats (*t* = 2.870, df = 14, *p* = 0.0123; independent samples *t*-test; Fig. 2B).

### Effect of crhbp knockdown on cocaine self-administration

**Fig. 3.**
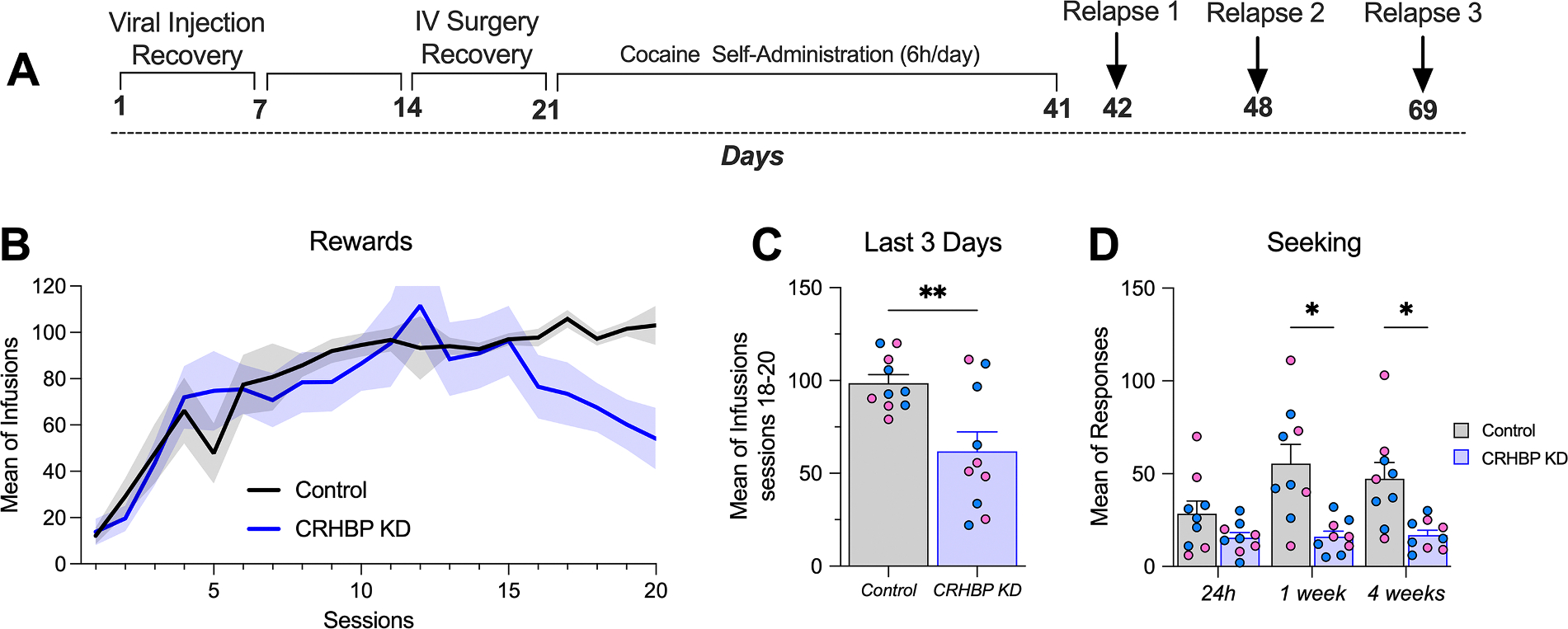
Cocaine self-administration. (A) Experimental timeline for cocaine self-administration experiments. (B) Average daily cocaine infusions for CRHBP KD animals and controls across 20-day exposure period. (C) The means of infusions for only the last 3 days (days 18–20) of cocaine self-administration. (D) The means of responses in reinstatement sessions 24 h, 1 week, and 4 weeks after the final day of cocaine self-administration. Data presented as mean ± SEM. *p < 0.05, **p < 0.01.

We evaluated the effects of *Crhbp* knockdown on intravenous cocaine self-administration across a 20-day period (Fig. 3A). Two-way repeated measures ANOVA with time and treatment as factors revealed a significant time × treatment interaction (F(19,342) = 2.396, *p* = 0.0010), a significant main effect of time (F(1,19) = 15.74, *p* < 0.0001), and a non-significant main effect of treatment (F(1,18) = 0.76, *p* = 0.392; Fig. 3B), indicating that reduced expression of *Crhbp* in the BLA blunted cocaine intake, specifically in the later sessions. The mean number of rewards during the last three sessions was significantly lower in the *Crhbp* knockdown group compared to controls (*t* = 3.200, df = 18, *p* = 0.0050; Fig. 3C). Two animals (one control, one shCrhbp) died during the protracted abstinence period and were excluded from the subsequent cue-induced drug-seeking experiments. In cue-induced drug-seeking tests conducted after abstinence periods of 24 h, 1 week, or 4 weeks, we found a significant time × treatment interaction (F(2,32) = 5.314, *p* = 0.0102), a significant main effect of time (F(1.983,31.7) = 5.83, *p* = 0.0064), and a significant main effect of treatment (F(1,16) = 12.30, *p* = 0.0029; Fig. 3D). Bonferroni’s multiple comparisons test showed that control rats exhibited significantly more active lever responses than knockdown rats at 1 week (adjusted *p* = 0.0150) and 4 weeks (adjusted *p* = 0.0241) post-self-administration, but not at 24 h (adjusted *p* = 0.3261).

### Effect of Crhbp knockdown on oxycodone self-administration

**Fig. 4.**
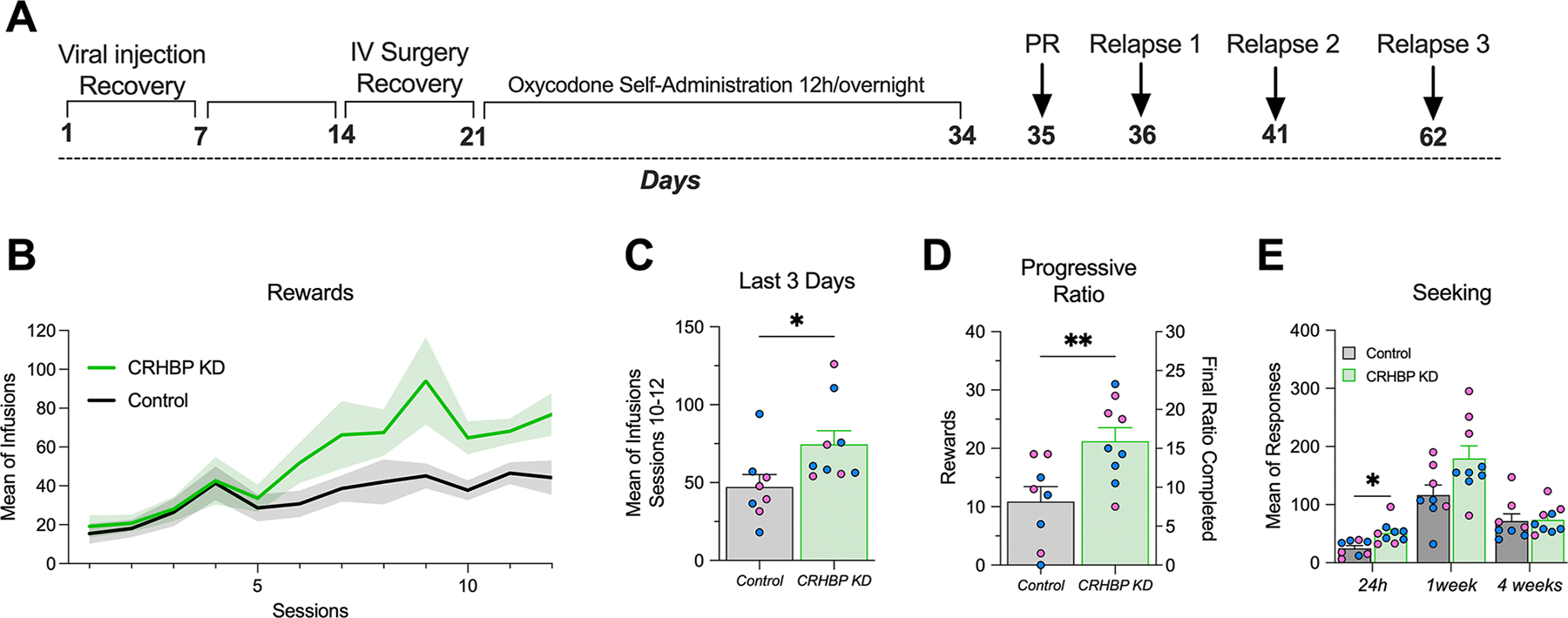
Oxycodone self-administration. (A) Experimental timeline for oxycodone self-administration experiments. (B) Average daily oxycodone infusions for CRHBP KD animals and controls across 12-day exposure period. (C) The means of infusions for only the last 3 days (days 10–12) of oxycodone self-administration. (D) The final ratio completed and averages of responses in the progressive ratio test for CRHBP KD animals and controls. (E) The means of responses in reinstatement sessions 24 h, 1 week, and 4 weeks after the final day of cocaine self-administration. Individual data points for male (blue circles) and female (pink circles) rats are shown. Data presented as mean ± SEM. *p < 0.05, **p < 0.01.

We next assessed the effects of *Crhbp* knockdown on intravenous oxycodone self-administration over a 12-day period (Fig. 4A). Two-way repeated measures ANOVA with time and treatment as factors revealed a significant time × treatment interaction (F(12,171) = 2.495, *p* = 0.0049), a significant main effect of time (F(2.882,41.07) = 13.24, *p* < 0.0001), and a non-significant main effect of treatment (F(1,15) = 2.956, *p* = 0.10; Fig. 4B), indicating that reduced expression of *Crhbp* in the BLA increased oxycodone intake over time. Given that this treatment effect emerged earlier than in the cocaine experiment, we terminated self-administration after 12 days and proceeded to additional testing. The mean number of rewards earned during the final three sessions was significantly higher in the *Crhbp* knockdown group compared to controls (*t* = 2.987, df = 15, *p* = 0.0092; Fig. 4C). Following the last self-administration session, oxycodone-trained rats underwent progressive ratio testing, where *Crhbp* knockdown rats exhibited greater motivation for oxycodone, completing significantly higher ratios than controls (*t* = 2.695, df = 13, *p* = 0.0184; Fig. 4D). In cue-induced drug-seeking tests conducted after abstinence periods of 24 h, 1 week, or 4 weeks, we observed a significant time × treatment interaction (F(2,30) = 4.483, *p* = 0.0198), a significant main effect of time (F(1.467,22.01) = 60.28, *p* < 0.0001), and a non-significant main effect of treatment (F(1,15) = 4.264, *p* = 0.0567; Fig. 4E). Bonferroni’s multiple comparisons test showed that *Crhbp* knockdown rats made significantly more active lever responses than controls at 24 h (adjusted *p* = 0.0155), but not at 1 week (adjusted *p* = 0.1164) or 4 weeks (adjusted *p* > 0.9999).

## Discussion

Our study reveals that corticotrophin-releasing hormone binding protein (CRHBP) in the basolateral amygdala (BLA) exerts substance-specific effects on addiction-related behaviors in rats, with knockdown reducing cocaine self-administration and cue-induced drug seeking while enhancing oxycodone intake, motivation, and early drug seeking. These findings underscore CRHBP’s complex modulatory role within the neurocircuitry of substance use disorders (SUDs), challenging a uniform function across drug classes and highlighting its potential as a therapeutic target.

The selective expression of *Crhbp* in BLA somatostatin-positive (*Sst*+) interneurons, as confirmed by snRNA-seq and RNAscope, aligns with its proposed role in modulating local inhibitory circuits. The BLA integrates emotional and reward-related signals, and *Sst*+ interneurons are known to regulate principal neuron excitability [37], influencing drug-seeking behaviors [38].

Our observation that *Crhbp* knockdown blunts cocaine intake and drug seeking suggests it may enhance inhibitory tone in cocaine-trained rats, counteracting the hyperexcitability linked to stimulant exposure [39]. This is consistent with human genetic data associating a *Crhbp* variant with improved cocaine cessation outcomes [26], implying that reduced *Crhbp* function might dampen cocaine-seeking circuits, possibly by altering corticotrophin-releasing factor (CRF) availability or signaling.

Conversely, the increased oxycodone self-administration, progressive ratio breakpoints, and 24-hour drug seeking in *Crhbp* knockdown rats suggest a facilitatory role in opioid reinforcement. This divergence could stem from *Crhbp’s* interaction with opioid-driven neuroadaptations in the BLA. Opioids like oxycodone reduce GABAergic inhibition in the BLA via μ-opioid receptor activation on interneurons [40], leading to disinhibition of principal neurons and increased excitability. *Crhbp* knockdown may exacerbate this effect, potentially by altering CRF signaling, thereby amplifying excitatory drive and enhancing motivation for oxycodone reward, as observed in our increased self-administration and progressive ratio breakpoints. The effects of *Crhbp* knockdown in the BLA reveal distinct temporal profiles: sustained reductions in cocaine self-administration and drug seeking persist at 1 and 4 weeks, whereas enhanced oxycodone intake and drug seeking peak at 24 h, show a trend at 1 week, and dissipate by 4 weeks. Research suggests that CRF modulates stress and anxiety during withdrawal across drug classes [41], offering a potential mechanism for these differential durations, possibly tied to prolonged stimulant-related adaptations versus transient opioid-driven changes.

These opposing outcomes resonate with prior preclinical work on *Crhbp* in other SUDs. For instance, CeA *Crhbp* knockdown reduces ethanol intake in dependent rats [22], paralleling our cocaine findings and suggesting a conserved role in mitigating negative reinforcement across some drug classes. However, our oxycodone results diverge from a lack of *Crhbp* association with opioid dependence in humans [27], possibly reflecting regional (BLA vs. CeA) or methodological (self--administration vs. genetic association) differences. The CRF-CRHBP system’s complexity, where CRHBP can both sequester CRF and facilitate its signaling via receptor interactions [42], may underlie these substance-specific effects, with cocaine and oxycodone differentially recruiting BLA circuits.

Our findings extend beyond CRHBP’s direct CRF modulation, implicating broader BLA network dynamics. The BLA’s ability to integrate emotional and reward-related signals is facilitated by its extensive anatomical connections with key brain regions involved in addiction. It maintains bidirectional connections with the prefrontal cortex (PFC), which is crucial for decision-making and inhibitory control, and with the hippocampus, which supports contextual memory formation [43–45]. The BLA’s projections to the nucleus accumbens (NAc) are critical for reinforcement and reinstatement [46,47], and *Crhbp* knockdown might alter these outputs in a drug-dependent manner. For cocaine, reduced BLA drive could weaken NAc dopamine signaling, blunting reward salience, while for oxycodone, heightened BLA activity might enhance NAc opioid responses, boosting motivation. Progressive ratio data further support this, as *Crhbp* knockdown rats showed greater effort for oxycodone, consistent with heightened incentive motivation [48]. Furthermore, as part of the extended amygdala, the BLA interacts with the central nucleus of the amygdala (CeA) and the bed nucleus of the stria terminalis (BNST), mediating stress and negative emotional states during withdrawal [49,50]. These connections allow the BLA to modulate both the rewarding and aversive aspects of drug use, influencing drug-seeking behaviors.

Several limitations of this study warrant consideration. Although snRNA-seq provided cell type-specific insights, the small number of *Crhbp*-expressing interneurons captured from the BLA (~45 cells) reflects both their low abundance and the technical limitations of isolating rare populations from this anatomically restricted region. Despite this, the clear overlap with *Sst* provides confidence in the biological relevance of these findings. While the viral knockdown approach proved to be tissue-specific and efficient, we cannot completely exclude potential off-target effects of the shRNA. Although we included both male and female Wistar rats, our study was not statistically powered to detect potential sex differences in *Crhbp* manipulation effects. This is a notable constraint, as sex differences in the CRF system and drug responses are well-documented. For instance, females exhibit heightened CRF receptor sensitivity and stress responses in the amygdala [51], which could influence *Crhbp’s* role in modulating cocaine and oxycodone reinforcement. Additionally, we and others showed that female rats self-administer more cocaine than males under long-access conditions [33,52], suggesting sex-specific vulnerabilities that our sample size may not capture. Similarly, research on oxycodone indicates females consume more than males in intravenous [36] and oral self-administration paradigms [53], potentially linked to differences in opioid system regulation [53], though these effects may vary by strain [35].

Furthermore, our focus on BLA *Crhbp* manipulation without targeting the CeA leaves questions about regional specificity unresolved, particularly given the CeA’s established role in ethanol withdrawal and stress responses [41]. Finally, while snRNA-seq identified *Sst*+ interneurons as a key site of *Crhbp* expression, the functional interactions between *Crhbp* and other BLA cell types, such as principal neurons or other interneuron populations, remain unexplored. These gaps highlight the need for future studies with larger, sex-balanced cohorts and broader regional analyses to fully elucidate *Crhbp*’s role in addiction.

These results carry translational implications. The divergent effects of *Crhbp* knockdown, reducing cocaine self-administration and reinstatement while enhancing oxycodone intake and motivation, suggest that therapeutic strategies targeting *Crhbp* may need to be drug-specific, potentially involving downregulation for cocaine use disorder and upregulation or stabilization for opioid use disorder. Clinical development of CRF1 receptor antagonists has proven challenging, with trials for various disorders [54–57] largely failing due to low efficacy, safety concerns, poor bioavailability, and rapid dissociation from the CRF1 receptor. As an alternative, pharmacological agents that indirectly modulate CRHBP activity or CRF signaling, such as those targeting its expression or downstream pathways, could be promising, though their efficacy may hinge on BLA-specific delivery.

Future studies should explore *Crhbp’s* downstream signaling (e.g., CRF receptor subtypes), sex differences, and the effects of prolonged abstinence periods to better capture chronic drug exposure dynamics.

In conclusion, *Crhbp* in the BLA modulates cocaine and oxycodone self-administration and reinstatement in opposing directions, reflecting substance-specific neuroadaptations. These findings advance our understanding of amygdalar contributions to SUDs and highlight *Crhbp* as a nuanced player in the balance of reward and negative reinforcement, offering a foundation for tailored therapeutic interventions.

## Supplementary Material

MMC2

MMC1

Supplementary materials

Supplementary material associated with this article can be found, in the online version, at doi:10.1016/j.addicn.2025.100221.

## Data Availability

Data will be made available on request.
