# Supplementary figures and images for: Corticotrophin-releasing hormone binding protein in the basolateral amygdala: divergent roles in cocaine and opioid addiction like behaviors
Source: Addict Neurosci. Author manuscript; Available in PMC 2026 Jul 3. (PMC13327722; doi:10.1016/j.addicn.2025.100221)

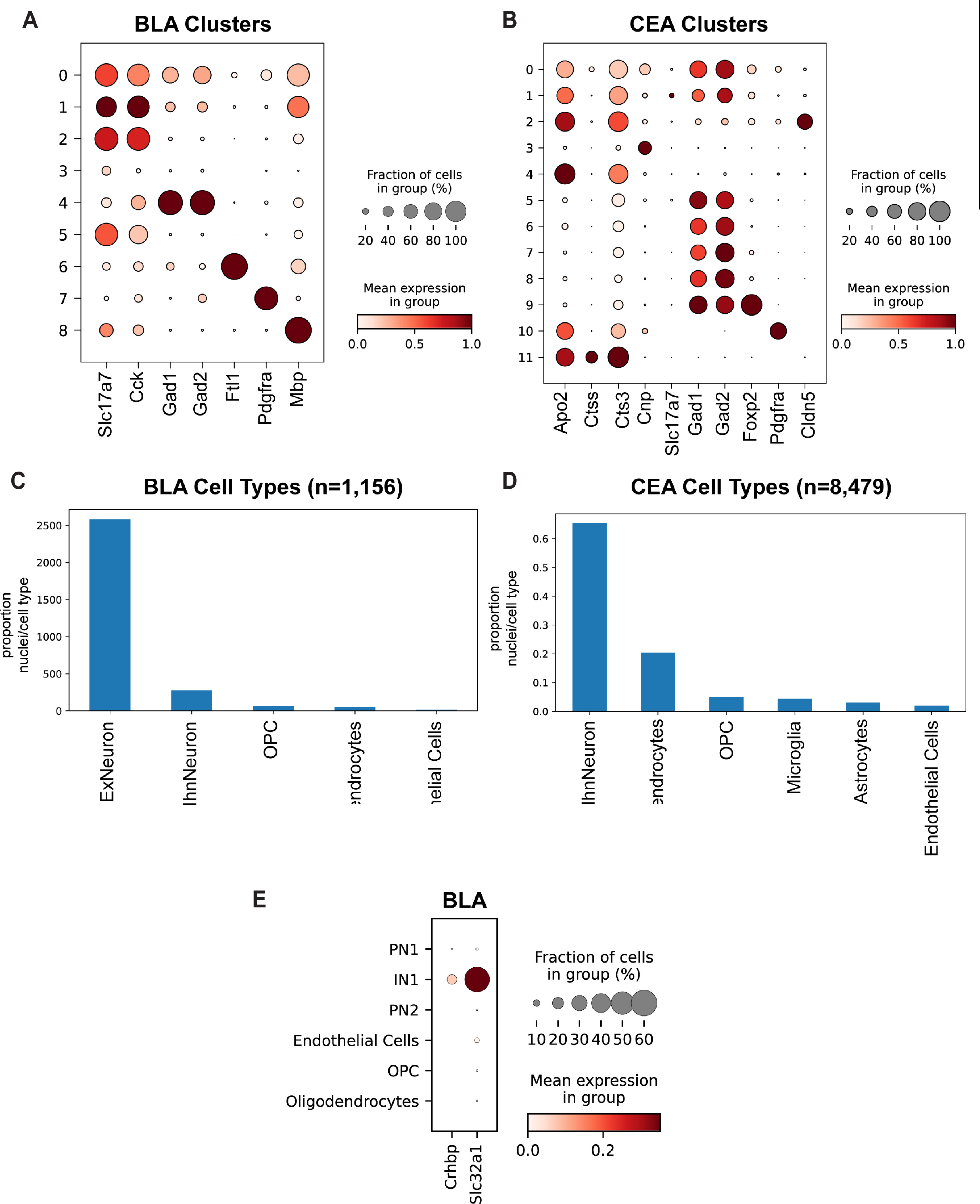

Supplement: MMC2 [file NIHMS2181387-supplement-MMC2.jpg]

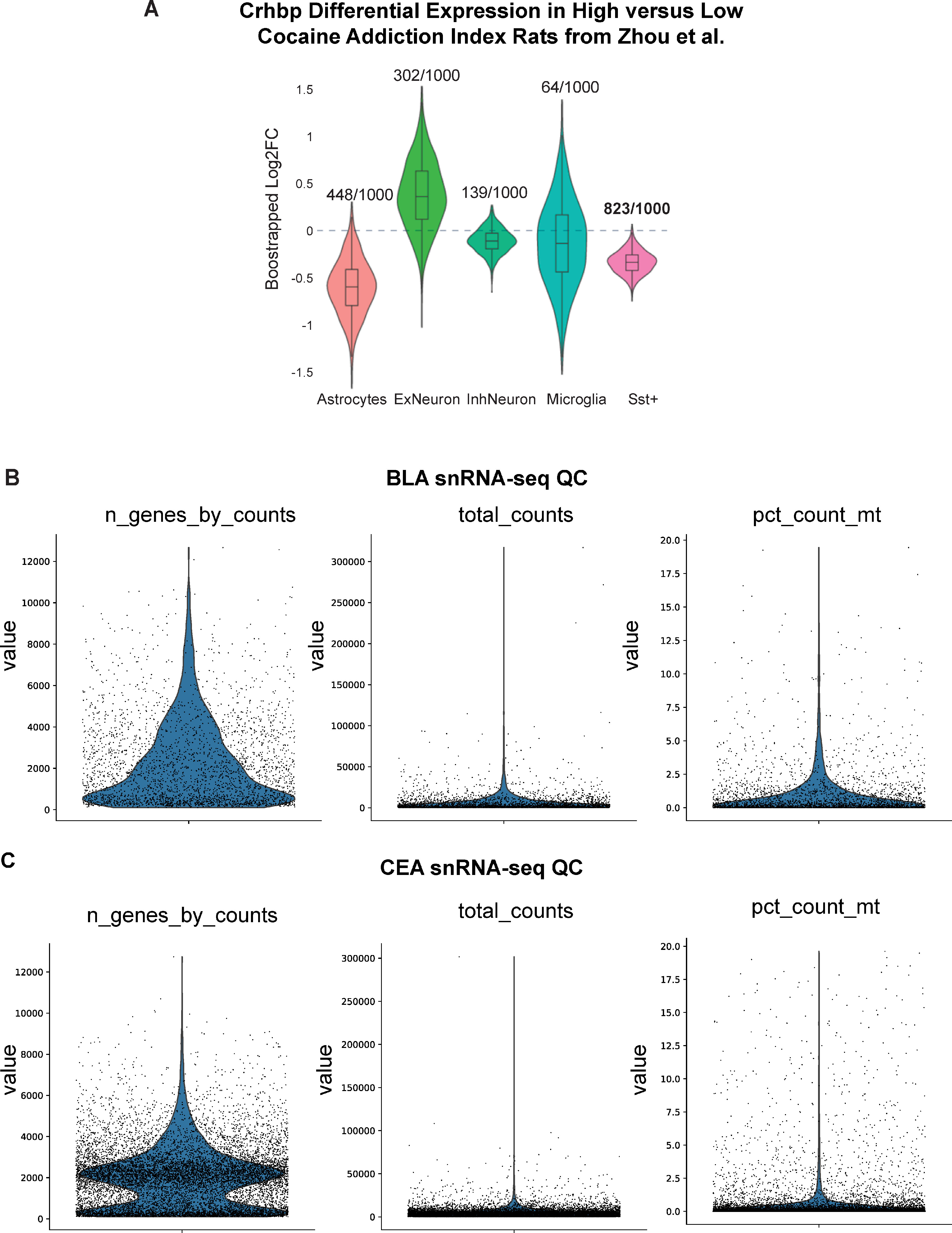

Supplement: MMC1 [file NIHMS2181387-supplement-MMC1.jpg]
